# In vitro activity of therapeutic antibodies against SARS-CoV-2 Omicron BA.1, BA.2 and BA.5

**DOI:** 10.1038/s41598-022-16964-z

**Published:** 2022-07-23

**Authors:** Franck Touret, Cécile Baronti, Boris Pastorino, Paola Mariela Saba Villarroel, Laetitia Ninove, Antoine Nougairède, Xavier de Lamballerie

**Affiliations:** Unité Des Virus Émergents (UVE: Aix-Marseille University - IRD 190 - Inserm 1207), Marseille, France

**Keywords:** Microbiology, Virology

## Abstract

The replacement of the Omicron BA.1 variant of SARS-CoV-2 by the BA.2 and the rapid growth of the BA.5 sub lineage, which have both different sets of mutations in the spike glycoprotein, alters the spectrum of activity of therapeutic antibodies currently licensed in the European Union. Using clinical strains of the Omicron BA.2 and BA.5 variants, we compared the neutralising power of monoclonal antibodies against the Omicron BA.1, BA.2 and BA.5 variants, using an ancestral strain (lineage B.1, D614G) and a Delta variant strain as reference. Sotrovimab/Vir-7831 is less active against BA.2 than against BA.1 (fold change reduction ~ 1,4) and even less active against BA.5 (fold change reduction ~ 2.7). Within the Evusheld /AZD7442 cocktail, Cilgavimab/AZD1061 is more active against BA.2 and BA.5 than against BA.1 (fold change increase ~ 32), whilst the very low activity of Tixagevimab/AZD8895 against BA.1 is not enhanced against BA.2 nor BA.5. In total, compared to BA.1, the activity of the Evusheld/AZD7442 is significantly improved against BA.2 while BA.5 is intermediate but closer to BA.2.

## Main

**Figure 1:**
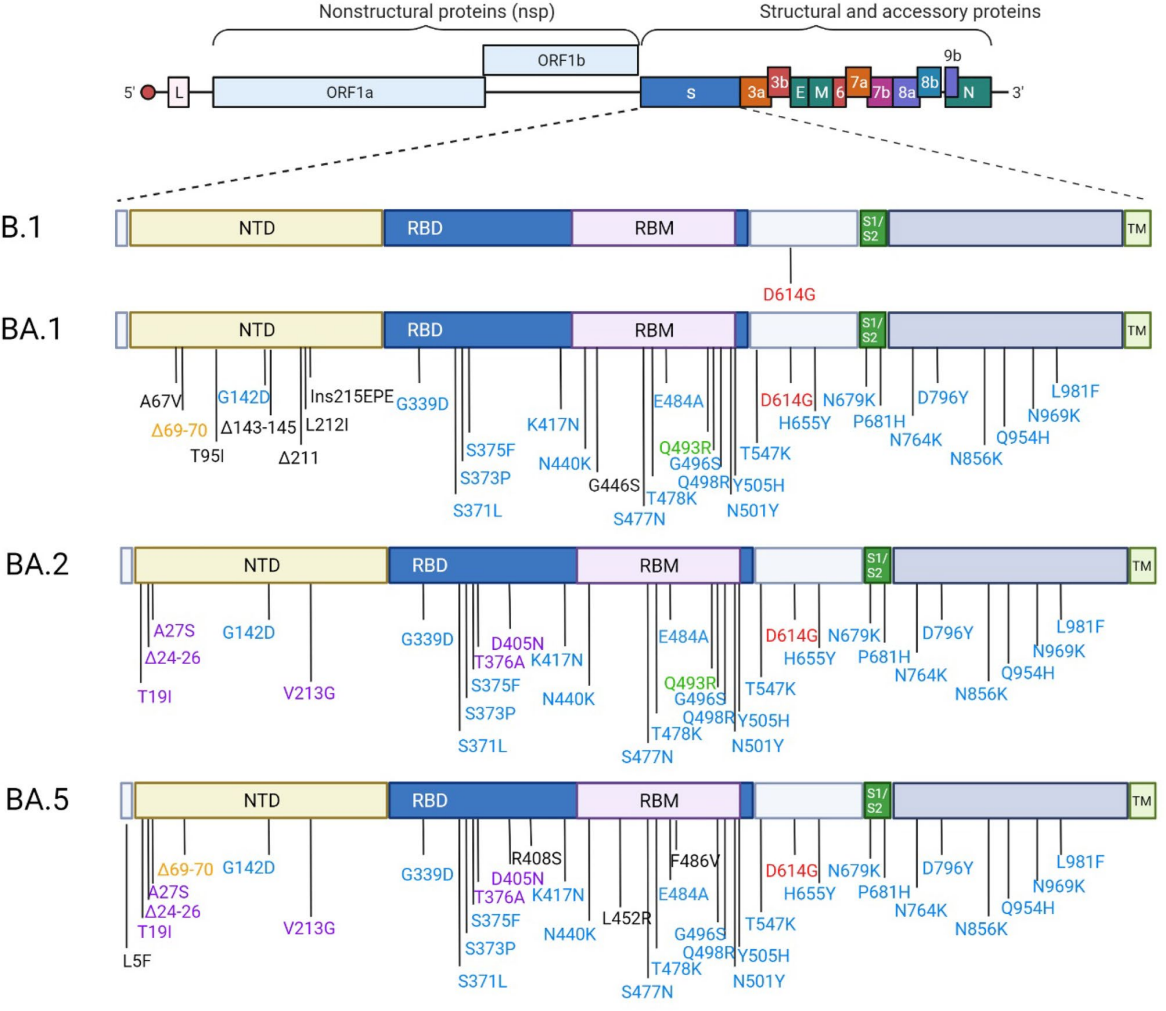
Spike substitutions in SARS-COV-2 variants Omicron BA.1, BA.2 and BA.5 compared to the ancestral strain B.1. Omicron BA.1, BA.2 and BA.5 sequences used for the representation are the one from the strains used in this study BA.1:EPI_ISL_7899754, BA.2: EPI_ISL_9426119 and BA.5: EPI_ISL_12852091.Red color indicates the mutation that is present in all strains. The blue color indicates the mutations which are common to BA.1, BA.2 and BA.5. The orange color indicates the mutations which are common to BA.1/BA.5. The purple color indicated the mutations which are common to BA.2/BA.5. This figure was created with BioRender.com*.*

The severe acute respiratory syndrome coronavirus 2 (SARS-CoV-2) emerged in China in late 2019 and then spread rapidly, causing the first pandemic of the twenty-first century. Since then, epidemic spread has been sustained by the continued emergence of new variants that combine increased transmissibility^1^ and antigenic shift^2^. We are currently witnessing the replacement of the Omicron BA.1 variant by a new sub lineage, BA.2, which also emerged in South Africa in late 2021. Omicron BA.2 has fewer mutations than BA.1 in the spike glycoprotein, some of which are shared with BA.1 and others are original^3^ (Fig. 1). In 2022 two new sub lineage, BA.4 and BA.5, emerged from the BA.2 lineage in South Africa^4^. Both BA.4/BA.5 share the same mutations in the spike glycoprotein and mainly differ from BA.2 regarding the 69-70del, L452R which was present in the Delta variant, F486V and the reversion to the original amino acid at Q493^4^(Fig. 1). These new mutation patterns have the potential to alter the activity of therapeutic monoclonal antibodies currently in clinical use.

In the current study, we tested the neutralising activity of therapeutic antibodies against clinical strains of the BA.1, BA.2 and BA.5 sub lineages of the B.1.1.529 Omicron variant, using the BavPat1 European ancestral strain (lineage B.1, D614G) and a Delta variant (B.1.617.2) as reference. We tested therapeutic antibodies currently in use that have been shown to retain neutralising activity against BA.1^5^. All target the spike Receptor Binding Domain (RBD)^6,7^ (Cilgavimab/AZD1061 and Tixagevimab/AZD8895, part of the Evusheld/AZD7442 cocktail) and more precisely the core region^6^ for Sotrovimab/Vir-7831.

**Figure 2:**
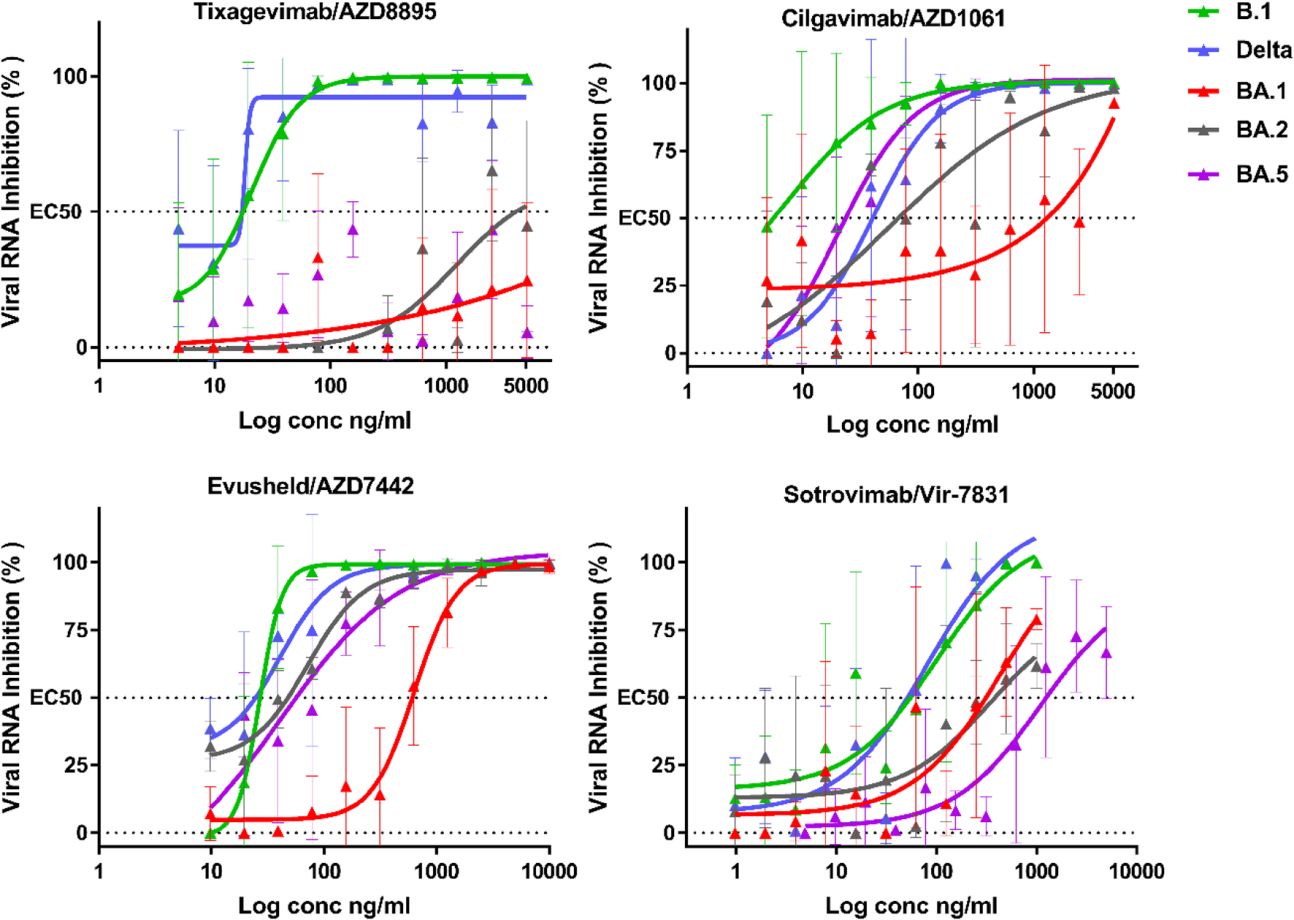
Dose response curves reporting the susceptibility of the SARS-CoV-2 BavPat1 D614G (B.1) ancestral strain,Delta BA.1 BA.2 and BA.5 variant to active therapeutic monoclonal antibodies Sotrovimab/Vir-7831,Tixagevimab/AZD8895, Cilgavimab/AZD1061 and Evusheld/AZD7742. Data presented are from one representative experiment. Data presented are from three technical replicates in VeroE6-TMPRSS2 cells, and error bars show mean ± s.d.

We used a standardised methodology for the evaluation of antiviral compounds based on the reduction of RNA yield^8–10^, which has been applied to SARS-CoV-2^11–15^. The assay was performed in VeroE6 TMPRSS2 cells and the amount of viral RNA in the supernatant medium was quantified by qRT-PCR 48 h post-infection to determine the 50% effective concentration (EC_50_) (Fig. 2).

Our results support previous studies reporting that Sotrovimab retains some neutralising activity against the BA.1 sub lineage in vitro^2,11,16,17^. In the case of the BA.2 variant (Table 1, Fig. 2), with an EC_50_ increasing from 46.0 (B.1) to 441.0(BA.2) ng/mL, we observe a decrease in neutralisation activity by a factor of ~ *9.6 (Table 1) compared to the ancestral B.1 strain, and ~ 1.4 compared to BA.1. This result is consistent with data from Vir Biotechnology using a pseudotype virus harboring all Omicron BA.2 spike mutations and with live viruses^3,18,19^. For BA.5 there is another decrease in Sotrovimab activity with an EC_50_ increasing to 858.2 ng/ml resulting in an 18.7 decrease in neutralization activity when compared to B.1 , ~ 2.7 compared to BA.1 and ~ 1.9 to BA.2. This loss of activity have been recently reported using neutralization with BA.4/BA.5 spike pseudo-virus^20^.

**Table 1:** Activity of therapeutic antibodies against B.1, Delta and Omicron BA.1, BA.2 and BA.5 variants.

Antibody			Strain				
			BavPat B.1	Delta	BA.1	BA.2	BA.5
GSK/ Vir	Sotrovimab (vir-7831)	EC_50_	46.0	51.5	315.6	441.0	858.2
		MNU_50_	72.4	64.7	10.6	7.6	3.9
		fold-change	–	1.1	6.9	9.6	18.7
AstraZeneca	Cilgavimab (AZD1061)	EC_50_	19.2	40.3	1617.0	49.8	23.5
		MNU_50_	52.1	24.8	0.6	20.1	42.6
		fold-change	-	2.1	84.2	2.6	1.2
	Tixagevimab (AZD8895)	EC_50_	18.3	17.2	n.n	n.n	n.n
		MNU_50_	54.7	58.1	n.n	n.n	n.n
		fold-change	–	0.9	–	–	–
	Evusheld (AZD7442)	EC_50_	20.2	24.7	594.6	37.4	56.6
		MNU_50_	99.0	80.8	3.4	53.5	35.3
		fold-change	–	1.2	29.4	1.9	2.8

Interpolated EC_50_ values are expressed in ng/mL. For B.1, BA.1 and BA.2 strains, the EC_50_ is the mean of two independent experiment (n = 2), each including three replicates. (n.n: non-neutralising). MNU_50_: neutralizing capacity per treatment expressed in million units. One unit is defined as the amount of a given antibody needed to provide 50% neutralization of 100 TCID50 of a given strain. Doses refer to treatments authorized in the European Union (Sotrovimab: 500 mg IV^21^; AZD7442: 300 mg IM (Cilgavimab 150 mg + Tixagevimab 150 mg). Fold change reduction were calculated in comparison with the ancestral B.1 strain.

The neutralising activity of Tixagevimab is very low against both BA.1, BA.2 and BA.5 (EC_50_ > 5000 ng/mL, see Table 1). In contrast, Cilgavimab regains neutralizing power against BA.2 and BA.5 with an EC_50_ increasing only from 19.2 (B.1) to 49.8 ng/mL (BA.2) and 23.5 ng/ml (BA.5), which represents a very limited loss of neutralising activity (B.1/BA.2 ratio: ~ 2.6 and B.1/BA.5 ratio: ~ 1.2 Table 1). In comparison, a 84.2-fold B.1/BA.1 reduction in neutralisation activity was observed with this monoclonal antibody. In short, this indicates that Cilgavimab exhibited 32-fold greater activity against BA.2 compared to BA.1 in our assays. This could be due to the absence in the BA.2 and BA.5 RBD of the G446S mutation (Fig. 1), which is located in a region identified as critical for Cilgavimab neutralising activity^6^. When Cilgavimab was tested in combination with Tixagevimab, as proposed in the Evusheld therapeutic cocktail^22^, the EC_50_ shifted from 20.2 (B.1) to 37.4 ng/mL (BA.2), *i.e.* a 1.9-fold decrease in neutralisation activity when comparing BA.2 with B.1, but a 15-fold increase when comparing BA.2 with BA.1 (Table 1). Regarding BA.5 there is a slight loss of the cocktail activity when compared to BA.2 with a 1.4 fold decrease but there is still a 10- fold increase when comparing BA.5 to BA.1. For the BA.2 sub-variant these results are perfectly in line with recent studies with live viruses and different read-out techniques^3,19^. For BA.5 our finding have also been confirmed by results recently produced using the BA.5/BA.5 spike protein-pseudo virus^20^.

The analysis of our results should be done in the context of the actual treatments administered to patients at risk of developing severe forms of Covid-19. Sotrovimab is registered in the European Union for the early treatment of infections with a single intravenous injection of 500 mg and the Evusheld AZD7442 cocktail for the prophylaxis of infection with a single 300 mg dose (150 mg Tixagevimab + 150 mg Cilgavimab, IM administration) but a possibility of double-dose curative use (300 mg Tixagevimab + 300 mg Cilgavimab, IV injection) was left open. As previously described^5^, based on the EC_50_ values, we calculated the neutralizing capacity of each treatment expressed as MNU_50_ (Table 1). This allows a realistic comparison between treatments of the neutralization capacity against each variants.

For Evusheld/AZD7442, the restoration of Cilgavimab activity against BA.2 results in a significantly improved activity per treatment compared to BA.1 (53.5 MNU_50_ *vs* 3.4 MNU_50_). For BA.5 the activity is also conserved despite the small decrease with 35.3 MNU_50_ . When the activity of a 300 mg dose of Evusheld/AZD7442 is compared to that of a 500 mg dose of Sotrovimab, the advantage goes to Sotrovimab for the BA.1 variant (10.6 MNU_50_ *vs* 7.6 MNU_50_ (BA.2) and 3.9 MNU_50_ (BA.5), but to Evusheld/AZD7442 for the BA.2 (53.5 MNU_50_ *vs* 7.6 MNU_50_ for Sotrovimab) and the BA.5 variant (35.3 MNU_50_ *vs* 3.9 MNU_50_ for Sotrovimab). The latter result was due to a combination of increased activity of Evusheld /ZD7442 against both BA.2 and BA.5, but also slightly lower activity of Sotrovimab against BA.2 and BA.5 compared to BA.1 (7.6 (BA.2) and 3.9 (BA.5) *vs* 10.6 MNU_50_).

We conclude that Sotrovimab 500 mg retains partial neutralizing activity against BA.2 and BA.5 despite two successive steps of decreased activity that must be closely monitored to ensure that the 500 mg dose is sufficient to provide therapeutic benefit against Omicron BA.2 and BA.5. The activity of a 300 mg dose of Evusheld /AZD7442 against BA.1 is limited in vitro and in vivo^23^, leading to a recent FDA recommendation to use a 600 mg dose instead^24^. The restored activity of Cilgavimab against BA.2 and BA.5 allows Evusheld /AZD7442 to regain significant activity against this variant. If a dose of 600 mg becomes the norm, the expected activity against BA.2 would be in the order of 107 MNU_50_ and 71 MNU_50_ for BA.5 , which are close to the activity originally observed with a 300 mg treatment against the European B.1 variant (~ 99 MNU_50_). However, as the neutralising activity of Tixagevimab is not restored against BA.2 and BA.5, it remains to be assessed by in vivo experiments whether the combination of Cilgavimab and Tixagevimab is still relevant compared to Cilgavimab alone, and to what extent AZD7442 acts against BA.2 and BA.5 as a monotherapy or a combination of antibodies.

## Methods

### Cell line

VeroE6/TMPRSS2 cells (ID 100978) were obtained from CFAR and were grown in minimal essential medium (Life Technologies) with 7 0.5% heat-inactivated fetal calf serum (FCS; Life Technologies with 1% penicillin/streptomycin (PS, 5000 U mL^−1^ and 5000 µg mL^−1^ respectively; Life Technologies) and supplemented with 1% non-essential amino acids (Life Technologies) and G-418 (Life Technologies), at 37 °C with 5% CO_2_.

### Antibodies

Sotrovimab/ Vir-7831 was provided by GSK (GlaxoSmithKline). Cilgavimab and Tixagevimab (AstraZeneca) were obtained from hospital pharmacy of the University hospital of La Timone (Marseille, France).

### Virus strain

SARS-CoV-2 strain BavPat1 was obtained from Pr. C. Drosten through EVA GLOBAL (https://www.european-virus-archive.com/) and contains the D614G mutation.

SARS-CoV-2 **Delta** variant was isolated in May 2021 in Marseille, France. The full genome sequence has been deposited on GISAID: EPI_ISL_2838050. The strain, 2021/FR/0610, is available through EVA GLOBAL (www.european-virus-archive.com, ref: 001 V-04282).

SARS-CoV-2 **Omicron** BA.1 (B.1.1.529) was isolated the 1st of December in Marseille, France. The full genome sequence has been deposited on GISAID: EPI_ISL_7899754. The strain, called 2021/FR/1514, is available through EVA GLOBAL (www.european-virus-archive.com, ref: 001 V-04436).

SARS-CoV-2 **Omicron** BA.2 (B.1.1.529) strain hCoV-19/France/NAQ-HCL022005338701/2022 was obtained from Pr. B. Lina and the sequence is available on GISAID : EPI_ISL_9426119.

SARS-CoV-2 Omicron BA.5 strain hCoV-19/France/ARA-HCL022074071401/2022 was obtained from Pr. B. Lina and the sequence is available on GISAID : EPI_ISL_12852091.

All experiments with infectious virus were conducted in a biosafety level 3 laboratory.

### EC50 determination

One day prior to infection, 5 × 10^4^ VeroE6/TMPRSS2 cells per well were seeded in 100µL assay medium (containing 2.5% FCS) in 96 well culture plates. The next day, antibodies were diluted in PBS with ½ dilutions from 1000 to 0.97 ng/ml for Sotrovimab and from 5000 to 4.8 ng/ml for Cilgavimab, Tixagevimab and its combination Evusheld . The 5000 to 4.8 ng/ml range of dilution was also used for for Sotrovimab with the BA.5 variant due do its decrease in activity. Eleven twofold serial dilutions of antibodies in triplicate were added to the cells (25µL/well, in assay medium). Then, 25µL of a virus mix diluted in medium was added to the wells. Each well was inoculated with 100 TCID_50_ of virus which correspond here to a MOI at 0.002 as classically used for SARS-Cov-2^12^. Prior to the assay it was verified for each variant that with this MOI, viruses in the cell culture supernatants were harvested during the logarithmic growth phase of viral replication at 48 h post infection^9,10,12^. Four virus control wells were supplemented with 25µL of assay medium. Plates were first incubated 15 min at room temperature and then 2 days at 37 °C prior to quantification of the viral genome by real-time RT-PCR. To do so, 100µL of viral supernatant was collected in S-Block (Qiagen) previously loaded with VXL lysis buffer containing proteinase K and RNA carrier. RNA extraction was performed using the Qiacube HT automate and the QIAamp 96 DNA kit HT following manufacturer instructions. Viral RNA was quantified by real-time RT-qPCR (GoTaq 1-step qRt-PCR, Promega) using 3.8 µL of extracted RNA and 6.2 µL of RT-qPCR mix and standard fast cycling parameters, i.e., 10 min at 50 °C, 2 min at 95 °C, and 40 amplification cycles (95 °C for 3 s followed by 30 s at 60 °C). Quantification was provided by four 2 log serial dilutions of an appropriate T7-generated synthetic RNA standard of known quantities (10^2^ to 10^8^ copies/reaction). RT-qPCR reactions were performed on QuantStudio 12K Flex Real-Time PCR System (Applied Biosystems) and analyzed using QuantStudio 12 K Flex Applied Biosystems software v1.2.3. Primers and probe sequences, which target SARS-CoV-2 N gene, were: Fw: GGCCGCAAATTGCACAAT; Rev: CCAATGCGCGACATTCC; Probe: FAM-CCCCCAGCGCTTCAGCGTTCT-BHQ1. Viral inhibition was calculated as follow: 100* (quantity mean VC- sample quantity)/ quantity mean VC. The 50% effective concentrations (EC50 compound concentration required to inhibit viral RNA replication by 50%) were determined using logarithmic interpolation after performing a nonlinear regression (log(agonist) vs. response – Variable slope (four parameters)) as previously described^8,10,12–14^. All data obtained were analyzed using GraphPad Prism 7 software (Graphpad software).

## Data Availability

The data that support the findings of this study are available from the corresponding authors upon reasonable request.
